# Two Different Life-Threatening Cases: Presenting with Torticollis

**DOI:** 10.1155/2016/7808734

**Published:** 2016-11-13

**Authors:** Gülsüm Alkan, Melike Emiroğlu, Ayşe Kartal

**Affiliations:** ^1^Faculty of Medicine, Department of Pediatric Infectious Diseases, Selcuk University, Konya, Turkey; ^2^Faculty of Medicine, Department of Pediatric Neurology, Selcuk University, Konya, Turkey

## Abstract

Acquired torticollis can be the result of several different pathological mechanisms. It is generally related to trauma, tumors, and inflammatory processes of the cervical muscles, nerves, and vertebral synovia. Although upper respiratory tract and neck inflammation are common causes of acute febrile torticollis in children, diseases with as yet undefined relationships may also result in torticollis. This is the case of spinal arachnoid cyst and pneumonia.

## 1. Introduction

Congenital torticollis usually arises from muscular fibrosis and neurological or bony abnormalities. Acquired torticollis typically results from inflammatory processes of the cervical muscles, nerves, or vertebral synovia. Retropharyngeal abscesses and upper respiratory tract infections are the most common infections associated with torticollis [1, 2].

Benign paroxysmal torticollis is another common cause of acquired torticollis characterized by recurrent episodes of head tilt often accompanied by vomiting, pallor, irritability, or ataxia. It usually presents in the first few months of life and resolves without complications within 1–5 years [3].

## 2. Case  1

**Figure 1:**
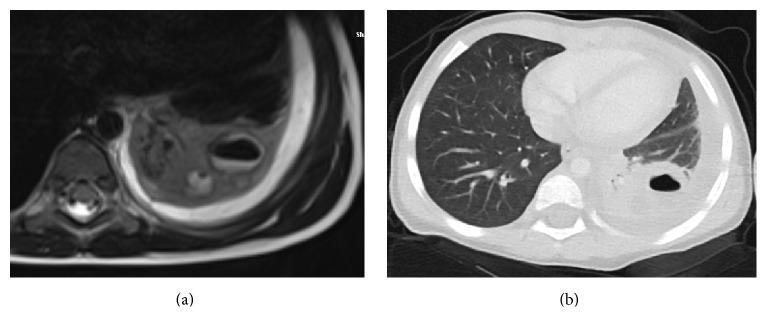
Thoracic vertebrae MR (a) and thorax CT (b) images: necrotizing pneumonia in the left lower lobe of lung.

A previously healthy, 2.5-year-old girl was referred to our hospital with limited neck motion, restlessness, and a mild fever over the three previous days. She was treated with ceftriaxone for the fever and subsequently referred to our facility after torticollis became evident. There was no history of trauma or cough. The physical examination revealed blood pressure 90/55 mm Hg, body temperature 36.2°C, pulse rate 110 beats/min, and respiratory rate 20/min. The remaining examination findings, including detailed neurological examination, were normal, with the exception of right-sided torticollis. Her laboratory data were as follows: white blood cell count (WBC) 19 × 10^3^/mm^3^, neutrophils 78%, erythrocyte sedimentation rate (ESR) 55 mm/hr, and C-reactive protein (CRP) 60 mg/dL. The magnetic resonance images (MRI) of the cervical and thoracic spine, taken in out center, were reevaluated; and necrotizing pneumonia in the left lower lobe of the lung and pleural effusion were revealed (Figure 1(a)). Necrotizing pneumonia was confirmed by thoracic computerized tomography (CT) (Figure 1(b)). Teicoplanin and ceftriaxone were initiated, after transthoracic aspiration, and methicillin resistant* Staphylococcus aureus* was grown. The torticollis improved gradually with antibiotic treatment, finally resolving completely, and she was discharged after five weeks of hospitalization.

## 3. Case  2

**Figure 2:**
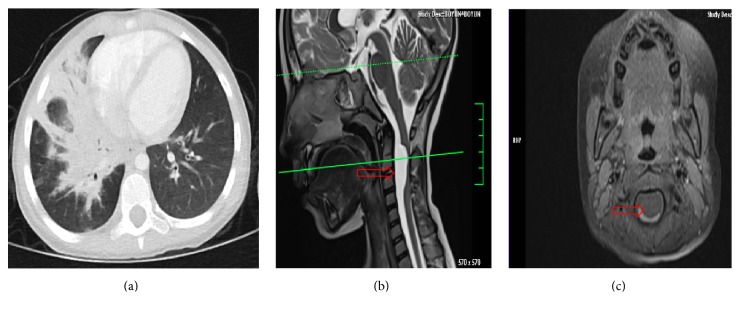
Thorax CT (b) and neck MR (b-c): right lung consolidation and cervical spine arachnoid cyst (arrow).

A 6-year-old female was admitted to our facility with persistent cough over the previous month and fever over the previous three days. Additionally, she complained of neck pain for the previous three months without torticollis. In another hospital, neck pain was thought to be due to the neck lymphadenopathy. The initial physical examination revealed temperature 39.5°C, pulse 120 beats/min, respiratory rate 25 breaths/min, and blood pressure 100/60 mm Hg. Crepitation was heard in the right lung, and enlarged cervical lymph nodes were observed bilaterally (>1 cm), with no redness or tenderness detected. All of the other systems were normal, with exception of torticollis on left side. Her initial WBC was 17 × 10^3^ cells/mm^3^ with 86% segments, ESR was 55 mm/hr, and CRP was 27 mg/dL. The chest X-ray and thoracic CT scan revealed consolidation in the middle and lower lobes of the right lung (Figure 2(a)). Retropharyngeal abscess was excluded and intravenous cefotaxime was given for the pneumonia. The torticollis was thought to be due to pleural irritation. This patient's conditions improved dramatically, and she was discharged after 10 days. One week later, she was readmitted with torticollis, but the detailed neurological examination was otherwise normal. MR scan of the neck was performed to rule out the retropharyngeal abscess, and an arachnoid cyst (2.5 × 1.5 cm^2^) was observed on the upper cervical spinal cord, markedly compressing the front of the spinal cord (Figures 2(b) and 2(c)). Surgery was planned immediately, before any neurological deficits developed.

## 4. Discussion

Inflammatory conditions of the upper respiratory tract and neck are common result in the unilateral muscle spasm responsible for the head posture [4]. Less common etiologies include cervical adenitis, osteomyelitis, tuberculosis, and upper lobe pneumonia. Tumors, ophthalmological problems, and central nervous system infections should also be considered, as well as Grisel's syndrome, nontraumatic subluxation of the atlantoaxial joint caused by inflammation of the adjacent tissues, and Chiari 1 malformation, that are rare but possible cause of headache, opisthotonus, and neck pain in children [1, 2, 5, 6]. Accurate differential diagnosis is required to correctly identify the cause and choose the right treatment.

Pneumonia presenting with torticollis is exceedingly rare, but seen must often in upper lobe pneumonia. To the best of our knowledge, isolated torticollis in pneumonia without any other symptoms has not been defined. Our patient had lower lobe pneumonia with no respiratory symptoms or auscultation findings. She was diagnosed incidentally with occult left lower lobe pneumonia via thoracic spinal MRI. Pneumonia may cause neck pain, stiffness, or torticollis secondary to compensatory muscle spasm, or referred pain. We believe that the pleural inflammation and irritation of the tracheobronchial tree caused the torticollis in our patient.

Spinal arachnoid cysts are less commonly in the children; they present with slowly progressive quadriparesis or neck pain. Surgical treatment is necessary for symptomatic spinal arachnoid cysts [7]. Spinal arachnoid cysts are rare in the pediatric population and found predominately in the thoracic and posterior spinal cord. Affected patients present with back pain, weakness, or gait instability [8]. In the literature, only 26 (17 children) cases had cysts anteriorly located in the cervical region, and the most frequent sign was quadriparesis. Previously, two pediatric patients presented with torticollis, neck pain, and paresis [9]. In our patient, we initially believed her neck pain was due to pneumonia as seen in first patient, but when her symptoms reoccurred, we looked for other causes. Fortunately, a cervical arachnoid cyst was detected before any neurological deficits develop.

Torticollis can be symptomatic of a life-threatening condition. Vital signs assessment, systemic examination, and neurological evaluation must be carefully and systematically performed in order to exclude serious illness, also taking into account rare conditions. Early diagnosis and treatment can prevent future complications.
